# Raloxifene May Have Further Benefits in Women on Hemodialysis

**DOI:** 10.5812/ijem.6580

**Published:** 2012-09-30

**Authors:** Roisin Worsley, Emorfia Gavrilidis, Jayashri Kulkarni

**Affiliations:** 1 Monash Alfred Psychiatry Research Centre, Monash University, Australia

**Keywords:** Raloxifene, Mood, Dialysis

Dear Editor,

Saito et al. report on the possible beneficial effects of raloxifene on bone metabolism in women on hemodialysis (1). We commend the authors on their investigation of treatment in this difficult group. Currently there are limited options available for osteoporosis management in women on hemodialysis which is a significant concern given that the fracture rate for these women is quadruple that of women in the general population (2). The role of postmenopausal osteoporosis in higher fracture rates in women on dialysis is increasingly recognized (2). The frequency of early menopause, menstrual disturbance and hypo-estrogenic states are also higher in women on dialysis. (3)

Menopause is a ‘window of vulnerability’ for depression in women, women on haemodialysis are particularly at risk of mental illness. A recent study found the prevalence of mental illness to be 30.3% in patients on hemodialysis (4). Mental illness, particularly depression has been found to be a predictor of reduced quality of life, increased morbidity and mortality (4). Whilst psychotropic medication can be used in hemodialysis patients, antidepressants and antipsychotic medications are independent risk factors for osteoporosis (5). Whether this risk is magnified in dialysis patients is not currently known.

Both estrogen therapy and SERMs have been investigated for their benefit on bone turnover in hemodialyis patients, they may well have further benefits. The mood enhancing effects of estrogen are well known (6). We have performed clinical trials showing improvement in psychotic symptoms with adjunctive transdermal estrogen therapy. Our recent dose finding study found raloxifene 120mg to also be effective at reducing positive and negative symptoms of psychosis in postmenopausal women. We are currently conducting larger clinical trials examining the effect of raloxifene on psychosis in pre and postmenopausal women. Given the mortality risks of mental illness and osteoporosis in women on hemodialysis it would be helpful if drug regimens that improve both conditions could be developed. Hopefully raloxifene will fulfill this role. We keenly await further data from Saito and Co. regarding the longer term effects of raloxifene in women on dialysis and particularly the impact on fracture risk.
